# Changes in plasma fatty acids profile in hyperketonemic ewes during early lactation: a preliminary study

**DOI:** 10.1038/s41598-022-21088-5

**Published:** 2022-10-11

**Authors:** Anastasia Lisuzzo, Filippo Fiore, Kevin Harvatine, Elisa Mazzotta, Michele Berlanda, Nicoletta Spissu, Tamara Badon, Barbara Contiero, Livia Moscati, Enrico Fiore

**Affiliations:** 1 Department of Animal Medicine, Production and Health, University of Padua, Viale dell’Università 16, 35020 Legnaro, Italy; 2 Department of Veterinary Medicine, University of Sassari, 07100 Sassari, Italy; 3 Department of Animal Science, Pennsylvania State University, State College, PA 16801 USA; 4 Istituto Zooprofilattico Sperimentale delle Venezie, 35020 Legnaro, Italy; 5 Istituto Zooprofilattico Sperimentale dell’Umbria e delle Marche, 06126 Perugia, Italy

**Keywords:** Animal physiology, Diagnostic markers

## Abstract

The transition from late pregnancy to early lactation is characterized by marked changes in energy balance of dairy ruminants. The mobilization of adipose tissue led to an increase in plasma non-esterified fatty acids (NEFA) and β-hydroxybutyrate (BHB). The aim of this study was to analyze the total plasma fatty acids of healthy and hyperketonemic dairy ewes in early lactation through gas chromatography (GC) to evaluate metabolic alterations. An observational study was used with a cross-sectional experimental design. Forty-six Sarda dairy ewes were enrolled in the immediate post-partum (7 ± 3 days in milk) and divided into two groups according to serum BHB concentration: non-hyperketonemic group (n = 28; BHB < 0.86 mmol/L) and hyperketonemic group (n = 18; BHB ≥ 0.86 mmol/L). A two-way ANOVA included the effect of group and parity was used to evaluate differences in fatty acids (FA) concentrations. A total of 34 plasma FA was assessed using GC. 12 out of 34 FA showed a significant different between groups and 3 out of 34 were tended to significance. Only NEFA concentration and stearic acid were influenced by parity. The results may suggest possible links with lipid metabolism, inflammatory and immune responses in hyperketonemic group. In conclusion, GC represents a useful tool in the study of hyperketonemia and primiparous dairy ewes might show a greater risk to develop this condition.

## Introduction

The transition from late pregnancy to early lactation is characterized by marked changes in endocrine and metabolic status of dairy ruminants. Energy requirements increase during this period as demands shift from fetal development and uterine metabolism to milk production^1,2^. The increased nutrient requirements and reduced feed intake results in negative energy balance (NEB)^3,4^. The NEB presents with different degrees of severity, but the nadir usually occurs within the first 10 days in milk (DIM)^5^.

The NEB associate with a reduced glucose concentration led to fatty acid (FA) mobilization from adipose tissue. The consequent increase of non-esterified fatty acids (NEFA) may be oxidized in the liver to produce energy. However, when there is a lack of glucose precursor, the amount of NEFA exceed the liver capacity and/or an alteration of tricarboxylic acid cycle realize, ketone bodies such as acetoacetate, β-hydroxybutyrate (BHB) and acetone are produced by NEFA^6–8^. Hepatic FA uptake can also outpace oxidation capacity leading to hepatic steatosis. Serum or plasma NEFA and BHB have become accepted biomarkers of excessive NEB. However, the first parameter denotes fat mobilization rate, whereas BHB reflects lipid oxidation^1,3,9^. Serum BHB is also the gold standard to diagnose ketosis with a threshold value greater or equal to 0.86 mmol/L in absence of clinical signs used to identify subclinical ketosis or hyperketonemia in sheep^10–12^.

Ketosis is a metabolic disorder affecting ewes regardless of farming purpose that may negatively influence animals’ health and production similar to that characterized in dairy cows. Investigation of ketosis in dairy ewes are mainly focused on pregnancy ketosis or ovine pregnancy toxaemia rather than lactational ketosis. Nonetheless, dairy ewes with high genetic merit for milk production experience an intense and prolonged NEB in early lactation^3,13,14^. Generally, risk of developing subclinical or clinical ketosis is increased as parity number increases^15^. However, animals at first parity have a lower social status and additional requirements for growth^16,17^ which also impact NEB.

In this study we hypothesize that animals affected by hyperketonemia during first days after calving have a modified plasma FA profile that is also modified by parity. For this reason, our aim was to perform a preliminary study to analyze plasma FA profile of healthy and hyperketonemic dairy ewes using GC for evaluating the metabolic alterations related to hyperketonemia.

## Results

### Main characteristics and biochemical parameters

Descriptive statistics about age, parity, DIM, milk production, and body condition score (BCS) of overall data and divided by groups were reported in Table 1. The low and high BHB groups did not differ in age, parity, DIM, BCS, or milk production; whereas serum BHB, NEFA, and glycemia were modified (*p*-value < 0.0001, = 0.030, and < 0.001, respectively) (Table 2). Moreover, NEFA also differed between groups in primiparous animals (0.35 mEq/L in hyperketonemic group vs 0.08 mEq/L in non-hyperketonemic group, SEM = 0.06; *p*-value = 0.026).

**Table 1:** Group descriptive data as means ± standard deviation and as median (first quartile; third quartile).

Item	Non-Hyperketonemic group	Hyperketonemic group	Overall data
Number of animals	28	18	46
Age (years)	4.7 ± 1.6	4.4 ± 1.9	4.6 ± 1.7
	4 (4; 6)	4 (3; 6)	4 (3; 6)
Parity	3.0 ± 1.5	2.8 ± 1.5	2.9 ± 1.5
	3 (2; 4)	2.5 (1; 4)	3 (2; 4)
Primiparous (n)	4	7	11
Secondiparous (n)	6	5	11
Multiparous (n)	18	8	24
DIM^1^	5.3 ± 0.7	4.5 ± 0.3	4.8 ± 0.4
	5 (4; 9)	4.8 (4; 10)	5 (4; 9)
Milk Production	1.26 ± 0.2	1.23 ± 0.1	1.24 ± 0.2
(Kg/day)	1.25 (1.23; 1.27)	1.23 (1.22; 1.24)	1.24 (1.22; 1.27)
BCS^2^	3.1 ± 0.7	2.8 ± 1.1	2.9 ± 0.9
	3 (2.5; 3.5)	3 (2; 3.5)	3 (2.5; 3.5)

^1^Days in milk.

^2^Body condition score.

**Table 2:** Characterization of ewes categorized as non-hyperketonemic group with low BHB or hyperketonemic group with high BHB (BHB > 0.86 mmol/L).

Parameters	Non-Hyperketonemic (n = 28)	Hyperketonemic (n = 18)	SEM	*p*-value
BHB (mmol/L)^1^	0.63	1.35	0.24	< 0.0001
Age (years)	4.6	4.6	0.21	0.402
Parity	3.2	2.3	1.49	0.707
DIM^2^	4.9	4.1	0.94	0.815
BCS^3^	3.0	2.6	0.19	0.130
Production (Kg/day)	1.25	1.22	0.05	0.827
NEFA^4^ (mEq/L)	0.12	0.19	0.02	0.030*
Triglycerides (mg/dL)	16.0	16.1	1.51	0.980
Cholesterol (mg/dL)	52.3	59.7	3.06	0.110
Glycemia (mg/dL)	75.1	60.6	2.68	< 0.001
Urea (mmol/L)	6.31	7.21	0.41	0.131
Total protein (g/L)	61.5	61.2	1.20	0.843
Albumin (g/L)	40.6	40.8	0.80	0.859
ALT^5^ (U/L)	16.3	16.6	0.72	0.773
AST^6^ (U/L)	122.7	130.4	7.50	0.468
LDH^7^ (U/L)	1075.8	1193.7	52.7	0.122
GGT^8^ (U/L)	88.6	76.4	5.37	0.117
ALP^9^ (U/L)	155.7	163.1	17.2	0.762

^1^β-Hydroxybutyrate.

^2^Days in milk.

^3^Body condition score.

^4^Non-esterified fatty acids.

^5^Alanine amino-transferase.

^6^Aspartate amino-transferase.

^7^Lactate dehydrogenase.

^8^γ-Glutamyl-transferase.

^9^Alkaline phosphatase;

*Significant interaction of the parity class (*p*-value = 0.026).

### Total plasma fatty acids profile

A total of 34 FA were identified in the plasma samples (Table 3) including 3 medium chain FA (MCFA; 8:0, 10:0, and 12:0), 23 long chain FA (LCFA; 14:0, 14:1 ω 5, 15:0, 16:0, 16:1 ω 9, 16:1 ω 7, 18:0, 18:1 ω 9, 18:1 ω 7, 18:2 ω 6, 18:3 ω 6, 18:3 ω 3, 18:4 ω 3, 20:0, 20:1 ω 9, 20:1 ω 7, 20:2 ω 6, 20:3 ω 9, 20:3 ω 6, 20:4 ω 6, 20:3 ω 3, 20:4 ω 3, and 20:5 ω 3), and 8 very-long chain FA (VLCFA; 22:0, 22:1 ω 9, 22:2 ω 6, 22:4 ω 6, 22:5 ω 3, 22:6 ω 3, 24:0, and 24:1 ω 9). Plasma total LCFA concentration was increased in hyperketonemic group (*p*-value = 0.003), whereas total MCFA and VLCFA concentrations tended to be reduced and increased, respectively, in the same group. Within specific FA, fifteen were different between groups: 1 MCFA, 7 LCFA, and 4 VLCFA with a *p*-value ≤ 0.05, and 2 LCFA, and 1 VLCFA with a 0.05 < *p*-value ≤ 0.10. There was an interaction with parity for 18:0 (Table 4), which was different in primiparous animals (25 mg/dL in hyperketonemic group vs 11.5 mg/dL in non-hyperketonemic group, SEM = 2.30; *p*-value = 0.014). The total plasma fatty acid (TPFA) concentration was significantly influenced by group (*p*-value = 0.004), as well as saturated (SFA), mono-unsaturated (MUFA), and unsaturated (UFA) fatty acids (*p*-value = 0.002, 0.006, and 0.011, respectively). Moreover, SFA interacted with parity class (*p*-value = 0.035) due to a difference in primiparous (50.5 mg/dL in hyperketonemic group vs 30.9 mg/dL in non-hyperketonemic group, SEM = 4.01). Lastly, polyunsaturated fatty acids (PUFA) and the ratio between saturated and unsaturated fatty acids (SFA:UFA) tended to a statistical significant increased. Among PUFA, the PUFA omega 3 (ω-3) showed a statistical significant increment in hyperketonemic group (*p*-value = 0.015), as well as the ratio between PUFA omega 6 (ω-6) and ω-3 (ω-6:ω-3) (*p*-value = 0.026).

**Table 3:** Total plasma fatty acid (TPFA) profile expressed as mg/dL of plasma and percent of TPFA of ewes categorized as non-hyperketonemic group with low BHB or hyperketonemic group with high BHB (BHB > 0.86 mmol/L).

Fatty acids	Nomenclature	Non-Hyperketonemic (n = 28)	Hyperketonemic (n = 18)	SEM	*p*-value
**Medium chain fatty acids** (**MCFA**)		3.78 (5.0%)	3.20 (3.56%)	0.18	0.099
8:0	Caprylic acid	3.13 (4.18%)	2.76 (3.07%)	0.13	0.041
10:0	Capric acid	0.29 (0.39%)	0.27 (0.30%)	0.03	0.310
12:0	Lauric acid	0.37 (0.50%)	0.17 (0.19%)	0.09	0.963
**Long chain fatty acids (LCFA)**		68.0 (90.91%)	83.5 (92.78%)	4.59	0.003
14:0	Myristic acid	0.52 (0.70%)	0.43 (0.48%)	0.05	0.463
14:1 ω 5	Myristelaidic acid	0.35 (0.47%)	0.09 (0.10%)	0.31	0.991
15:0	Pentadecanoic acid	0.41 (0.55%)	0.39 (0.43%)	0.02	0.885
16:0	Palmitic acid	14.1 (18.85%)	16.5 (18.33%)	0.95	0.015
16:1 ω 9	Hypogeic acid	0.35 (0.47%)	0.36 (0.40%)	0.03	0.467
16:1 ω 7	Palmitoleic acid	0.31 (0.41%)	0.43 (0.48%)	0.04	0.006
18:0	Stearic acid	12.9 (17.25%)	18.8 (20.89%)	1.19	< 0.001*
18:1 ω 9	Oleic acid	10.8 (14.44%)	15.2 (16.89%)	1.49	0.007
18:1 ω 7	Vaccenic acid	0.99 (1.32%)	1.32 (1.47%)	0.12	0.039
18:2 ω 6	Linoleic acid	20.2 (27.00%)	21.0 (23.33%)	1.08	0.502
18:3 ω 6	γ-Linolenic acid (GLA)	0.58 (0.78%)	0.57 (0.63%)	0.04	0.518
18:3 ω 3	α-Linolenic acid (ALA)	0.97 (1.30%)	2.19 (2.43%)	0.64	0.065
18:4 ω 3	Stearidonic acid (SDA)	0.07 (0.10%)	0.09 (0.10%)	0.01	0.090
20:0	Arachidic acid	0.06 (0.08%)	0.04 (0.04%)	0.01	0.028
20:1 ω 9	Gondoic acid	0.03 (0.04%)	0.02 (0.02%)	0.01	0.131
20:1 ω 7	Paullinic acid	0.01 (0.01%)	0.01 (0.01%)	0.01	1.000
20:2 ω 6	Eicosadienoic acid	0.16 (0.21%)	0.13 (0.14%)	0.01	0.249
20:3 ω 9	Mead acid	0.13 (0.17%)	0.18 (0.20%)	0.02	0.155
20:3 ω 6	Dihomo-γ-linolenic acid (DGLA)	0.31 (0.41%)	0.29 (0.32%)	0.03	0.897
20:4 ω 6	Arachidonic acid	4.10 (5.48%)	4.53 (5.03%)	0.25	0.164
20:3 ω 3	Eicosatrienoic acid (ETE)	0.02 (0.03%)	0.02 (0.02%)	0.01	0.885
20:4 ω 3	Eicosatetranoic acid (ETA)	0.01 (0.01%)	0.01 (0.01%)	0.01	0.249
20:5 ω 3	Eicosapentaenoic acid (EPA)	0.62 (0.83%)	0.89 (0.99%)	0.06	0.004
**Very long chain fatty acids (VLCFA)**		3.06 (4.09%)	3.36 (3.73%)	0.17	0.062
22:0	Behenic acid	0.08 (0.11%)	0.08 (0.09%)	0.01	0.303
22:1 ω 9	Erucic acid	0.01 (0.01%)	0.01 (0.01%)	0.01	1.000
22:2 ω 6	Docosadienoic acid	0.03 (0.04%)	0.02 (0.02%)	0.01	0.375
22:4 ω 6	Adrenic acid (ADA)	0.50 (0.67%)	0.28 (0.31%)	0.07	0.007
22:5 ω 3	Decosapentaenoic acid (DPA)	1.09 (1.46%)	1.31 (1.46%)	0.07	0.014
22:6 ω 3	Docosahexaenoic acid (DHA)	0.66 (0.88%)	0.89 (0.99%)	0.08	0.014
24:0	Lignoceric acid	0.37 (0.50%)	0.40 (0.44%)	0.02	0.054
24:1 ω 9	Nervonic acid	0.33 (0.44%)	0.37 (0.41%)	0.03	0.015
TPFA^1^ mg/dL		74.8	90.0	4.69	0.004
SFA^2^ mg/dL		32.1 (42.96%)	39.8 (44.26%)	3.52	0.002*
UFA^3^ mg/dL		42.7 (57.09%)	50.2 (55.81%)	4.69	0.011
SFA^2^/UFA^3^		0.75	0.80	0.04	0.065
MUFA^4^ mg/dL		13.2 (17.65%)	17.8 (19.78%)	2.70	0.006
PUFA^5^ mg/dL		29.5 (39.40%)	32.4 (36.03%)	2.59	0.084
ω-3^6^ mg/dL		3.43 (4.59%)	5.39 (5.99%)	1.20	0.015
ω-6^7^ mg/dL		25.9 (34.67%)	26.9 (29.86%)	2.26	0.524
ω-6^7^/ ω-3^6^		7.59	6.61	0.40	0.026

^1^Total plasma fatty acids.

^2^Saturated fatty acids.

^3^Unsaturated fatty acids.

^4^Mono-unsaturated fatty acids.

^5^Polyunsaturated fatty acids.

^6^Fatty acids ω-3.

^7^Fatty acids ω-6.

*Significant interaction of the parity class.

**Table 4:** Fatty acids (mg/dL) significantly affected by parity class of ewes categorized as non-hyperketonemic group with low BHB or hyperketonemic group with high BHB (BHB > 0.86 mmol/L).

Fatty acids	Parity class*	Non-Hyperketonemic group (n = 28)	Hyperketonemic group (n = 18)	SEM	*p*-value
18:0	1	11.5^a^	25.0^b^	2.30	0.014
	2	15.2	17.5	2.24	
	3	11.9	13.8	1.51	
SFA	1	30.9^a^	50.5^b^	4.01	0.035
	2	36.5	36.9	3.90	
	3	29.0	32.1	2.63	

*1: primiparous; 2: secondiparous; 3: multiparous.

^a-b^Mean values in the same row which differ significantly.

## Discussion

Ketosis is associated to hyperketonemia, hypoglycemia and uremia^18^. The ketone bodies derive from partially oxidation of NEFA, which are mobilized from adipose tissue during NEB^19^. In our study, serum NEFA concentration increased in hyperketonemic group, particularly within primiparous animals. These findings suggest that the hyperketonemic group had a greater lipid mobilization in early postpartum compared to healthy group, especially primiparous animals. The amount of mobilized NEFA is related to triacylglycerols mobilization from adipose tissue^20^. In our study, blood triglycerides concentration did not change and its concentration in both groups was within the normal range (< 100 mg/dL) according to Van Saun^19^. However, triacylglycerols are broken down to NEFA and glycerol in adipose tissue and released in blood stream^20,21^, justifying the absence of alteration in this biochemical parameter.

If the amount of NEFA entering in the liver exceeds its capacity to oxidize them, then NEFA are converted to triglycerides. Hepatic triglycerides are exported by lipoproteins^4,22^. Among lipoproteins, there are the very low-density protein (VLDL) which transport cholesterol to tissues^23^. Low cholesterol concentration suggests a reduced capacity for liver export of triglycerides resulting in their accumulation in hepatic tissue^19^. According to Van Saun^19^, cholesterol concentration should be more than 70 mg/dl. In our study, both groups were under this cut-off (52.3 mg/dL in non-hyperketonemic group and 59.7 mg/dL in hyperketonemic group). This may suggest potential risk for develop of hepatic lipidosis in both groups, especially in primiparous due to their greater concentration of serum NEFA^16^. The reference range for glycemia is between 50 and 85 mg/dL^19^. In our study, both groups were within the normal range (75.1 mg/dL in non-hyperketonemic group and 60.6 mg/dL in hyperketonemic group). However, serum glucose was reduced in hyperketonemic group compared to non-hyperketonemic group. Urea is generated in the liver by the urea cycle for the detoxification of ammonia^24^. The normal range for urea is between 2.86 and 7.14 mmol/L^25^. An increase of its concentration is associated to protein mobilization during NEB^26^. In our study, urea concentrations were within the normal range and suggested the absence of protein mobilization in hyperketonemic group.

Ketosis is often associated to liver dysfunctions including hepatic lipidosis or fatty liver^19^. Furthermore, these metabolic disorders are related to subclinical inflammation^27^. Hyperproteinemia derives from an increase of albumin, globulin, or both. The only cause of hyperalbuminemia is dehydration, while hyperglobulinemia derives from inflammatory states^24^. In our study, the total protein and albumin concentrations were within the normal range expressed by Van Saun^19^ with the consequent hypothesis that the hyperketonemic group was not affected by inflammatory status.

Enhanced lipolysis due to NEB leads to an increase in NEFA concentration, but also to a change in TPFA profiles^4,28,29^. Increased NEFA is associated with in reduction in mammary de novo FA synthesis and increase of milk FA derived from adipose tissue mobilization during early lactation. The adipose tissue of dairy cows releases mainly LCFA, predominantly palmitic (16:0), stearic (18:0), oleic (18:1 ω 9), and linoleic (18:2 ω 6) acids^22,28,30^. In the present study, TPFA profile confirmed the same pattern in dairy ewes. In fact, these four FA represented about 78% of total lipid plasma in both groups. The mobilization of adipose tissue should be considered as a homeorhetic response to support lactation^5^. However, hyperketonemic group appeared to have greater lipid mobilization in early postpartum as suggested by the increase in NEFA and LCFA, including palmitic, stearic, and oleic acids.

Similar to NEFA, stearic acid increased in hyperketonemic group, especially in primiparous animals. Greater NEFA in primiparous compared to multiparous was previously reported in the literature. In fact, primiparous show behavioral and physiological differences compared to multiparous due to their lower social status and additional energy requirement for growth^16,17,31^. Furthermore, parity number also influences angiogenesis, turnover, and survival of mammary gland cells during lactation. Growth and mammary gland cells proliferation continues into early lactation of animals at first parity^32,33^. The major increase in stearic acid in hyperketonemic primiparous ewes may suggest that these animals were more predisposed to develop a lactational hyperketonemic state due to greater lipid mobilization to support lactation, growth, and tissue remodeling.

The tendency for a decrease in plasma MCFA may suggest a lower plasma concentration of MCFA during hyperketonemia. Indeed, the study of Xue et al.^21^ observed that the MCFA concentration in hepatic tissue of feed restricted ewes which develop ketosis were reduced compared to a control group. This result was related to a greater β-oxidation of MCFA compared to LCFA. The reduction of plasma MCFA may also be related to rumen microbiome and fermentation. In fact, MCFA may also be adsorbed by the rumen wall and from duodenal flow of ruminal microbial FA^34^. Among the MCFA, only caprylic acid (8:0) showed a reduction in hyperketonemic group in the study of Liu et al.^35^ wherein was evidenced a statistical significant reduction of SFA including caprylic acid on serum of dairy cows affected by ketosis. The reduction of MCFA may indicate its utilization for β-oxidation in hepatocytes because all animals received the same TMR.

The ω-3 α-linolenic acid (ALA; 18:3 ω 3), eicosapentaenoic (EPA; 20:5 ω 3), and docosahexaenoic (DHA; 22:6 ω 3) acids may modulate gene expression through the peroxisome proliferator-activated receptors (PPARs). Specifically, these FA may induce the activation of PPARα^29^ which regulates peroxisomal FA metabolism including transport and β-oxidation. Peroxisomes are a cellular organelle engaged primarily in VLCFA oxidation^36,37^. The reduced plasma concentration of lignoceric (24:0) and nervonic (24:1 ω 9) acids were related to an increase in VLFCA oxidation by peroxisomes^38^. In this study, plasma ALA, EPA, and DHA increased in hyperketonemic ewes. However, both lignoceric and nervonic acids increased in the same group. These results may indicate that VLCFA were not primarily used for β-oxidation compared to MCFA and that their increase during fat mobilization leads to greater concentrations of VLFCA in milk as in the study of Fiore et al.^14^ on dairy ewes’ milk.

The increase in plasma palmitic and stearic acids may influence animals’ metabolism through the induction of pyruvate carboxylase’s gene transcription with a subsequent alteration of tricarboxylic acid cycle^39^. According to Liu et al.^35^, the serum of ketotic cows had higher concentrations of UFA and MUFA, and lower concentrations of SFA and SFA:UFA ratio. A similar pattern was also seen in cows’ milk due to an impaired functioning of the tricarboxylic acid cycle^40^. The plasma lipid fraction of dairy ewes used in this study showed an increase for SFA and SFA:UFA ratio in contrast to dairy cows; whereas UFA, MUFA, and PUFA showed the same pattern. The difference may be due to a distinct BHB cut-off between the two species or a difference in severity of the condition.

Increasing palmitoleic acid (16:1 ω 7) by venous infusion in sheep reduced intramuscular adipocytes size and lipid content and tended to restore insulin sensitivity^41^. Studies conducted on bovine adipocytes showed a reduction of lipogenesis (around 50%) and increase in β-oxidation consequent to palmitoleic or vaccenic (18:1 ω 7) acids addition^42,43^. Vaccenic acid derives from diet or biosynthetic pathways through the elongation of palmitoleic acid^41,43,44^. Furthermore, palmitoleic acid upregulates GLUT4 receptor in adipocytes and hepatocytes^41^. An increase in these FA in hyperketonemic group may suggest an increase in the elongase enzyme and their use to manage energy requirement, glucose uptake and insulin sensitivity during hyperketonemia.

Arachidic acid (20:0) is a SFA whose concentration differs between bovine tissues such as muscle and kidney adipose tissue^45–47^. In the study of Newsome et al.^45^ investigating the digital cushion of dairy cows, adipocytes size and concentration of arachidic acid were positively related to BCS. In the present study, the two groups did not differ in BCS. However, the NEB induces fat mobilization from functional fat reserves such as the digital cushion^45^. The reduction of arachidic acid in hyperketonemic group may be related to an increase of β-oxidation induced by palmitoleic and vaccenic acids.

The increase in plasma SFAs, UFAs, MUFAs, and PUFAs can alter inflammatory and immune responses due to their interaction with the toll-like receptors (TLRs), nuclear factor-κ B (NFκB), and cell membrane lipid profiles^14,29,48,49^.

Among the main LCFA mobilized from adipose tissue, palmitic and stearic acids were increased in hyperketonemic group according to the NEB status and early lactation stage^29,39,50^. These FA are important regulators of immune and inflammatory response. The addition of palmitic acid to cellular proteins (palmitoylation) induces a change of their structure and ability to bind lipid bilayers. This process is necessary to activate leukocyte receptors during immune response^29^. However, an increase of SFA in cellular membranes negatively influences its fluidity and consequently cellular functions^51^. Furthermore, palmitic and stearic acids may induce the expression of cytokines, chemokines, and their receptors enhancing the cellular inflammatory response other than increase in reactive oxygen species (ROS) production^14,29^. The activation of immune and inflammatory responses normally occurs in the periparturient period due to tissue damage and remodeling to support a new physiological state^5^. However, the increase of palmitic and stearic acids in hyperketonemic group denoted a more important alteration of these responses in this group. Palmitoleic and oleic (18:1 ω 9) acids can derive from palmitic and stearic acids, respectively, through the enzymatic activity of stearoyl-CoA desaturase^23,42^. Both these FA were related to immunosuppression and inhibition of lymphocyte functions in ketotic ewes^51^. Moreover, oleic acid was proposed as a predictor of subclinical ketosis in dairy cows^22^. These FA were both increased in hyperketonemic group suggesting alteration of immune response in hyperketonemic animals.

In addition to the SFAs and MUFAs previously cited, ω-3 FA also may play a role in inflammatory responses. The ω-3 are involved in eicosanoid metabolism and may be mobilized during inflammatory event^52^. A precursor of ω-3 is ALA, which must be provided by the diet^53^. In fact, dietary FA are extensively biohydrogenated in the rumen by microorganisms^44,54^. Different conjugated linolenic acids (CLnA) and ω-3 FA may be obtained from ALA through enzymatic activities. Among them, there are stearidonic (SDA; 18:4 ω 3), EPA, docosapentaenoic (DPA; 22:5 ω 3), and DHA acids^55,56^ whose concentrations were increased in the hyperketonemic group compared to non-hyperketonemic group. The ω-3 FA may be incorporated into cellular membranes as phospholipids and, after oxidized by cyclooxygenases and lipoxygenases to oxylipids, be involved in inflammatory response. Specifically, mediators derived from ω-3 are primarily anti-inflammatory or pro-resolving^57,58^. In this study, hyperketonemic group increased ω-3 without a change in concentration of ω-6 resulting in a reduction of ω-6:ω-3 ratio. This study presented a cross-sectional experimental design and therefore it is not possible to know exactly the previous state of the animals. Considering this design, the ewes might be affected by a previous inflammatory state that normally occurs during peripartum period. Inflammation might have redirected nutrients and energy facilitating the onset of hyperketonemia^5,8^. The possible previous inflammatory state led to the mobilization of ω-3 needed for their anti-inflammatory proprieties. Thus, the context observed at the time of sampling may represent the initial mobilization of ω-3 that have not yet been used, or may have already been used and reduced from a previous state.

Adrenic acid (ADA; 22:4 ω 6) is an ω-6 FA more concentrated in liver, vasculature, kidney, adrenal gland, and brain. It can be converted into arachidonic acid through β-oxidation, which play an important role in inflammatory responses^57,59,60^. The plasma ADA concentration increases in humans and mice affected by nonalcoholic fatty liver disease. The presence of greater concentration of ADA predisposes to an oxidative stress state due to an increased ROS production and modulated expression of antioxidant systems^61,62^. Specifically, ADA positively influences the expression of superoxide dismutase (SOD) and negatively influences the expression of glutathione peroxidase (Gpx) in a culture of human hepatocytes^61,62^. In the study of Radin et al.^1^ conducted during the transition period of dairy goats, primiparous showed a peak in Gpx:SOD ratio in the first days of lactation. This finding was associated to an oxidative stress at the onset of lactation with a consequent adaptive response to it. In this study, the reduction of ADA in hyperketonemic group might indicate its previous use to produce arachidonic acid for the inflammatory response. This hypothesis would agree with a possible previous inflammatory state in progress of resolution that contributed to redirect the energetic resources and predisposed the animals to the state of hyperketonemia. Moreover, the reduced levels of ADA suggest an increase in Gpx and a decrease in SOD with a consequence increase in Gpx:SOD ratio accordingly to an adaptive response at the onset of lactation.

Considering the present results of this preliminary study, further studies would be necessary to evaluate more animals and more flocks.

## Conclusion

The changes in plasma FA composition during early lactation hyperketonemia may help to understand metabolic adaptations of animals to a NEB state, confirming the GC as a useful tool in the study of this condition. The FA composition suggests possible relationships with lipid metabolism, inflammatory and immune responses that should be further investigated. Furthermore, a higher risk to develop hyperketonemia was suggested in primiparous ewes indicating that the management of high yielding dairy ewes need further investigation to improve and preserve animals’ health. Future studies would be necessary to better investigate metabolic alteration during the transition period in this species.

## Materials and methods

All the procedures related to animals were conducted according to Directive 2010/63/EU of the European Parliament and of the Council of 22nd September 2010 on the protection of animals used for scientific purposes (Article 1, Paragraph 1, Letter b) and the Italian legislation (D. Lgs. n. 26/2014, Article 2, Paragraph 1, Letter b). The study has received the approval of the Ethics Committee of Sassari University, Protocol number 128469/2019. This study was carried out in compliance with the ARRIVE guidelines.

### Animals

The study was carried out on a flock of high-yielding Sarda dairy sheep located in a commercial farm in the north of Sardinia (Italy). The flock consisted of 800 lactating ewes. All ewes were fed a total mixed ration (TMR) formulated for lactating sheep (40–50 kg of body weight—BW) with an average milk production of 1.2 kg/day for a standard lactation. The protein content was 15% DM and the energy value of the ration was 9.5 ME (Mj/kg DM). The ewes grazed 1 h/day natural pasture. Forty-six animals lambed twins and were enrolled in this study during the early post-partum period (7 ± 3 DIM), as the development of ketosis is more frequent between the sixth week before lambing and the first weeks of lactation^63–65^. The same animals were used for the study of Fiore et al.^14^ that investigated the changed in milk FA according to different levels of BHB. The BCS was evaluated by the same person on a scale of 1 to 5 points, with 1 being emaciated and 5 being extremely fat^66^. Data on age and parity were also acquired. Moreover, enrolled animals were submitted to a clinical examination by the Veterinarians of the University of Sassari (Italy) and were clinically healthy.

### Experimental design

An observational study was used with a cross-sectional experimental design. Blood was collected from the jugular vein for each animal using a vacutainer system. One aliquot was collected in a tube containing EDTA (5 mL; Terumo Venoject, Leuven, Belgium) and one was stocked in a tube containing clot activator (9 mL; Terumo Venosafe, Leuvel, Belgium). Blood BHB concentration was measured immediately using a portable digital reader (Abbott Precision Xtra™ meter, Oxon, UK) and blood ketone test strips (Abbott Precision Xtra™ Blood Ketone test strips, Oxon, UK) to ensure that the minimum number of animals with hyperketonemic state was reached. The blood was transported in a cold box to the laboratory of the University of Sassari (Italy) at 4 °C within 1 h.

The samples were centrifuged at 1750 g × 10 min using a centrifuge (Hettich® EBA 20 centrifuge, Stuttgart, DE) to obtain serum and plasma. Next, 250 µL of extracted plasma were added to 5 mg of pyrogallol into a 1.5 mL microcentrifuge tube and stirred until the pyrogallol was completely dissolved to prevent fatty acid oxidation. Samples were sent by overnight shipment on dry ice to University of Padua (Italy), at the Department of Animal Medicine, Production and Health (MAPS). Serum and plasma samples were stored at − 20 °C for biochemical analysis and GC analysis, respectively.

**Figure 1:**
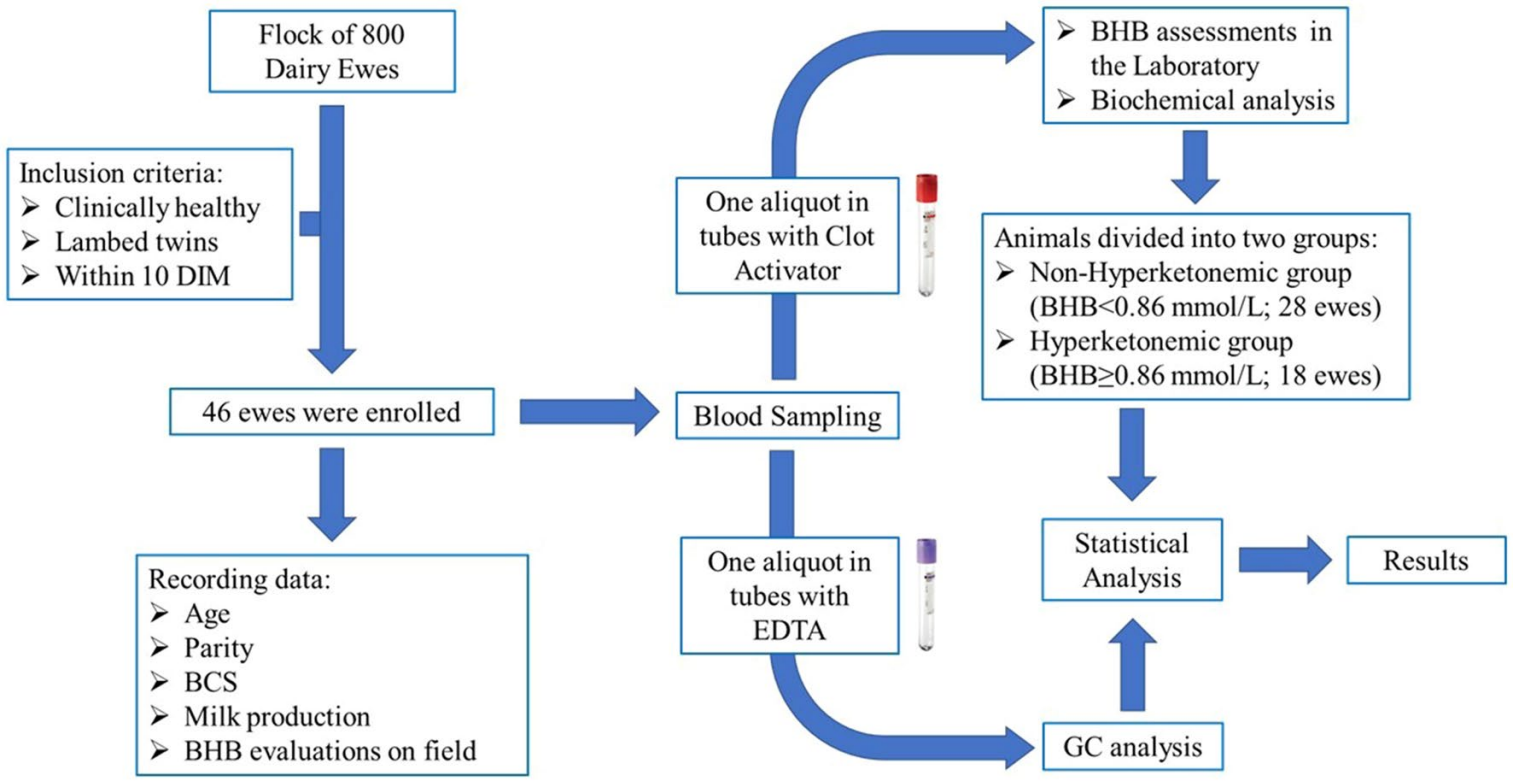
Flowchart of experimental design.

Experimental design, inclusion criteria and trial steps were described in Fig. 1.

### Blood analysis

Serum biochemistry was performed in the laboratory of the Experimental Zooprophylactic Institute of Umbria and Marche (IZSUM, Perugia, Italy) as described by the study of Fiore et al.^14^ using an automatic clinical chemistry analyzer (Konelab 200, Italy). Serum BHB concentrations were also measured using β-hydroxybutyrate Enzymatic Kinetics assay (Randox, Crumlin, UK; BHB, mmol/L). Serum NEFA concentration was determined with the NEFA RX Monza colorimetric method (Randox, Crumlin, UK). Glucose was measured using colorimetric test (Sclavo Diagnostic Dasit-Italy) and urea using a kinetic method (Sclavo Diagnostic Dasit-Italy).

Animals were grouped after blood analysis according to serum BHB concentration measured in the laboratory into two groups: non-hyperketonemic group (BHB concentration < 0.86 mmol/L; n = 28 ewes) and hyperketonemic group (BHB concentration ≥ 0.86 mmol/L; n = 18 ewes)^11^.

### Gas chromatography analysis

Plasma FA analysis by GC was performed by the laboratory of MAPS. The samples were directly methylation by 3 N methanolic HCL after addition of internal standards (9:0 and 17:0 triacylglycerols; 1 mg/mL for both internal standards) to perform quantification of FA.

Briefly, after addition of the methanolic HCL the samples were placed in an oven for one hour at 100 °C and then neutralized with a solution of potassium carbonate (K_2_CO_3_). During each step of lipid extraction, the vials containing the samples were insufflated with a stream of nitrogen to saturate the air present to limit fatty acid oxidation. Furthermore, the butylated hydroxytoluene (BHT) was added to hexane solvent to further limit oxidation. The resulting FA methyl esters were separated and quantified in split less mode by GC using a TRACE GC/MS (Thermo Quest, Milan, Italy) equipped with a flame ionization detector (FID) and a polar fused-silica capillary column (Capillary Column Omegawax, 30 m × 0.25 mm × 0.2 µm film). Helium was used as the carrier gas at a flow rate of 1 mL/min. A total of 34 FA was identified through comparison with an analytical reference standard consisting of a fatty acid mix (C6-C24 GLC Reference Standards; Nu-Check-Prep Inc., Elysian, MN, US) and a standard reference mix of PUFAs (PUFA No. 3, 47,085-U; Sigma-Aldrich, St. Louis, MO, US). A MS was also performed for fatty acids of doubtful identification. Data for plasma FA were calculated in mg/dL. The TPFA was calculated by adding identified FA minus the internal standard.

### Statistical analysis

The sample size was established with MedCalc for Windows ver. 19.4 (MedCalc Software, Ostend, Belgium). A minimum of 26 animals equally divided into two groups were necessary to recognize as statistically significant a difference greater than or equal to 0.3 mmol/L in BHB concentration^10^ according an α error of 0.05 and a power of 0.95. Statistical analysis was performed using the S.A.S. system software ver. 9.4 (SAS Institute Inc., Cary, North Caroline, USA) and R software ver. 4.0.3 implemented with “rcmdr” package (R Core Team, Vienna, Austria). The accepted *p*-value was ≤ 0.05, whereas a *p*-value between 0.05 and 0.1 was considered as a trend to significance.

Normal distributions of data were assessed by Shapiro–Wilk test before any analysis. One-way ANOVA was performed to evaluate statistical differences in BHB concentration, age, parity, and DIM; whereas BCS, milk production, biochemical parameters, and FA concentrations were assessed by two-way ANOVA. The first model included only the fixed effects of group, while the second model included the fixed effects of parity (3 level: primiparous, secondiparous, and pluriparous), group, and their interaction. The hypotheses of linear model on the residuals were graphically assessed. A post-hoc pairwise comparison among least squares means were performed using Bonferroni correction.

## Data Availability

The data presented in this study are available by sending an email to the corresponding author.
